# A Randomized Placebo-Controlled Trial of Intermittent Preventive Treatment in Pregnant Women in the Context of Insecticide Treated Nets Delivered through the Antenatal Clinic

**DOI:** 10.1371/journal.pone.0001934

**Published:** 2008-04-09

**Authors:** Clara Menéndez, Azucena Bardají, Betuel Sigauque, Cleofé Romagosa, Sergi Sanz, Elisa Serra-Casas, Eusebio Macete, Anna Berenguera, Catarina David, Carlota Dobaño, Denise Naniche, Alfredo Mayor, Jaume Ordi, Inacio Mandomando, John J. Aponte, Samuel Mabunda, Pedro L. Alonso

**Affiliations:** 1 Barcelona Center for International Health Research (CRESIB) and Department of Pathology Hospital Clinic, Institut d'Investigacions Biomedicas August Pi i Sunyer (IDIBAPS), Universitat de Barcelona, Barcelona, Spain; 2 The Manhiça Health Research Center (CISM), Maputo, Mozambique; 3 National Directorate of Health and National Malaria Control Program, Ministry of Health, Maputo, Mozambique; 4 National Institute of Health, Ministry of Health, Maputo, Mozambique; University of North Carolina at Chapel Hill, United States of America

## Abstract

**Background:**

Current recommendations to prevent malaria in African pregnant women rely on insecticide treated nets (ITNs) and intermittent preventive treatment (IPTp). However, there is no information on the safety and efficacy of their combined use.

**Methods:**

1030 pregnant Mozambican women of all gravidities received a long-lasting ITN during antenatal clinic (ANC) visits and, irrespective of HIV status, were enrolled in a randomised, double blind, placebo-controlled trial, to assess the safety and efficacy of 2-dose sulphadoxine-pyrimethamine (SP). The main outcome was the reduction in low birth weight.

**Findings:**

Two-dose SP was safe and well tolerated, but was not associated with reductions in anaemia prevalence at delivery (RR, 0.92 [95% CI, 0.79–1.08]), low birth weight (RR, 0.99 [95% CI, 0.70–1.39]), or overall placental infection (p = 0.964). However, the SP group showed a 40% reduction (95% CI, 7.40–61.20]; p = 0.020) in the incidence of clinical malaria during pregnancy, and reductions in the prevalence of peripheral parasitaemia (7.10% vs 15.15%) (p<0.001), and of actively infected placentas (7.04% vs 13.60%) (p = 0.002). There was a reduction in severe anaemia at delivery of borderline statistical significance (p = 0.055). These effects were not modified by gravidity or HIV status. Reported ITN's use was more than 90% in both groups.

**Conclusions:**

Two-dose SP was associated with a reduction in some indicators, but these were not translated to significant improvement in other maternal or birth outcomes. The use of ITNs during pregnancy may reduce the need to administer IPTp. ITNs should be part of the ANC package in sub-Saharan Africa.

**Trial Registration:**

ClinicalTrials.gov NCT00209781

## Introduction

Every year 50 million women become pregnant in areas where malaria is endemic, and at least half of them live in Africa [1]. Malaria during pregnancy is associated with maternal and foetal morbidity and mortality through maternal anaemia, low birth weight (LBW) and premature delivery [2]–[6]. Thus, prevention of malaria during pregnancy is a public health priority, particularly in sub-Saharan Africa.

Currently, prevention of malaria in pregnancy in Africa relies on intermittent preventive treatment (IPTp) and insecticide treated nets (ITNs) [1]. ITNs are associated with significant health benefits for both the mother and the newborn in sub-Saharan Africa [7]. IPTp entails the provision of an antimalarial at treatment doses irrespective of the presence of parasites or symptoms. The currently recommended regimen for IPTp is at least 2 treatment courses of SP given from the 2^nd^ trimester onwards at least one month apart [1].

Although IPTp and ITNs have shown separately to be efficacious in reducing the harmful effects of malaria during pregnancy, limited information exists on the safety and efficacy of both interventions together [8], [9].

IPTp, is now being implemented throughout most malaria endemic areas of Africa, and has become somewhat controversial. This is fuelled by two elements; on the one hand, the spread of SP resistance, and the other, the overlap in some areas of malaria transmission and high HIV prevalence. IPTp with SP is not recommended to HIV-positive women receiving cotrimoxazole prophylaxis or antiretroviral drugs [10]. Thus, although HIV-positive women have an increased risk of malaria [11], current recommendations may fail to provide them adequate protection.

In order to evaluate the safety and efficacy of two intermittent doses of SP in women of all parities and regardless of HIV status, who had been given a long-lasting ITN (LLITN) through the antenatal clinic (ANC), we carried out a randomised, double blind, placebo-controlled trial of IPTp in Mozambican pregnant women. This information should help guide policy towards the rational use of control tools for malaria prevention in pregnancy.

## Methods

The protocol for this trial and supporting CONSORT checklist are available as supporting information; see Checklist S1 and Protocol S1.

### Study area and population

The study was undertaken at the Centro de Investigação em Saúde da Manhiça (CISM) in Manhiça District, southern Mozambique. A demographic surveillance system covering 36 000 inhabitants is carried out by the CISM and constitutes the study area. Adjacent to the CISM is the Manhiça District Hospital (MDH), a 110 bed health facility. The characteristics of the area have been described in detail elsewhere [12]. Perennial malaria transmission with some seasonality is mostly attributable to *P falciparum*. *Anopheles funestus* is the main vector, and the estimated entomological inoculation rate for 2002 was 38 infective bites per person per year. Data on the efficacy of SP in children in this area showed a therapeutic efficacy rate of 83%, with an *in vivo* parasitological sensitivity of 78.6% at day 14 [13]. Eighty percent of pregnant women have an institutional delivery. During the study, malaria control in pregnancy relied exclusively upon case management.

### Study design

This double blind, individually randomised, placebo controlled trial had the primary objective of estimating the additive protective effect of two-dose IPTp with SP to that of ITNs on LBW prevalence. Based on previous estimates in the area, a LBW prevalence of 20% and an estimated 25% reduction to 15% in the presence of ITNs [14], 411 women per group were needed to show a lack of difference between the two groups with a confidence interval of 7 (8% to 22%), at the 5% level of significance with a 80% statistical power. The study protocol was approved by the National Mozambican Ethics Review Committee, and the Hospital Clinic of Barcelona Ethics Review Committee.

### Enrolment and interventions

From August 2003 to April 2005 pregnant women were enrolled at the MDH ANC if their gestational age was ≤ 28 weeks, they did not report allergies to sulpha drugs, and they were permanent residents of the CISM study area. After written informed consent was obtained, the lowest available study number was assigned. A computer-generated sequential list contained the study numbers linked to treatment identification letters, randomly ordered in blocks of 10. Tablets of SP or placebo, identical in shape and colour, were stored in 10 bottles labelled only with a single treatment identification letter. Women were randomised to receive 3 tablets of SP (1500 mg sulphadoxine/75 mg pyrimetamine) or placebo. The project health nurse administered study drugs to women with at least 12 weeks of gestational age. Doses were given twice from the second trimester, at least one month apart. Regardless of their gestational age, all women at recruitment received a LLITN. Assessment of gestational age was made by bimanual palpation of the fundal height. Haemoglobin and the rapid plasma reagin test (RPR, Syphacard, Wellcome, USA) were assessed as part of the routine antenatal care. In accordance to the National Programme for HIV control, women were offered voluntary counselling and testing; HIV positive women and their babies were given Nevirapine prophylaxis and referred for further clinical follow-up.

### Follow-up

Plastic photo cards of mother and child facilitated identification at every contact throughout the study (from recruitment until 8 weeks postpartum). A health facility-based, passive surveillance system was established at the MDH. At each consultation, a standardized questionnaire was completed documenting signs and symptoms. Blood films were prepared for malaria parasite examination and the packed cell volume (PCV) measured if there was a history of fever in the preceding 24 hrs, or the axillary temperature was ≥37.5°C. Clinical malaria episodes were treated with chloroquine or SP in the first and subsequent trimesters, respectively, for uncomplicated malaria, and parenteral quinine for severe malaria.

At delivery, venous blood was collected by venipuncture from the mother and the umbilical cord, and two thick blood smears and a filter paper prepared for haematological and parasitological determinations. Newborns were weighed on a digital scale, accurate to the nearest gram. The gestational age was assessed by the Dubowitz's method [15]. A placental biopsy was collected and two impression smears and blood placed onto filter paper were prepared from a placental biopsy sample.

Eight weeks after delivery a capillary blood sample was collected from the mother and the infant for parasite and haematological determinations. The axillary temperature was measured and the weight of the baby recorded.

ITN use was assessed by asking the women if they had slept under the LLITN the night before.

### Laboratory Methods

Thick and thin blood films were stained and read according to standard, quality-controlled procedures [16], [17]. PCV was measured in a microcapillary tube after centrifugation. Blood samples were centrifuged and the plasma and erythrocyte pellets stored at −70°C.

HIV serostatus was assessed using a rapid test (Determine, Abbot Laboratories, USA), and positive results confirmed using Unigold rapid test (TM HIV, Trinity Biotech, Ireland).

Tissue samples were collected from the maternal side of the placentas and placed into 10% neutral buffered formalin. Biopsies were processed and stained following standard procedures [18]. Impression smears from the placenta, were stained with Giemsa and read following standard procedures [19].

### Data Management, Statistical Methods and Definitions

Data were analysed by intention-to-treat (ITT) analysis whereby all randomised women were included regardless of whether or not they had received the intervention and the number of doses.

The risk of the first or only episode of clinical malaria between recruitment and 8 weeks postpartum, or censoring due to withdrawal or death, was estimated using Cox regression models. The protective effect (PE) of SP was estimated from the hazard ratio (HR) as PE = 100(1-HR) %.The effect of time on efficacy since dose 1 was estimated using time versus treatment interaction in a Cox time-dependent model. In the secondary analyses, crude and adjusted (for parity, HIV status and baseline variables) prevalences were calculated using the Risk Ratio. Likelihood Ratio tests were calculated to evaluate the interaction between the intervention group and parity, or HIV status. Differences in prevalence were estimated with the χ^2^ test and proportions with the Fisher's exact test. Continuous values were evaluated with the non-parametric Wilcoxon test. Data analysis was performed using Stata 8.2 (Stata Corporation, College Station, TX, USA).

Malaria infection was defined as the presence of asexual P. falciparum parasites of any density in a blood smear. A clinical malaria episode was defined as the latter plus an axillary temperature ≥37.5°C. The duration of a malaria episode was estimated as 28 days. Overall and severe anaemia were defined as a PCV lower than 33% and 21%, respectively.

Placental infection was classified according to a previous definition [20]. Any placental infection was defined as the presence of parasites and/or pigment in the histological examination, and/or in the impression smear.

Women with a multiple delivery (twins or triplets) were also included in the analysis.

## Results

### Baseline characteristics of study women

**Figure 1:**
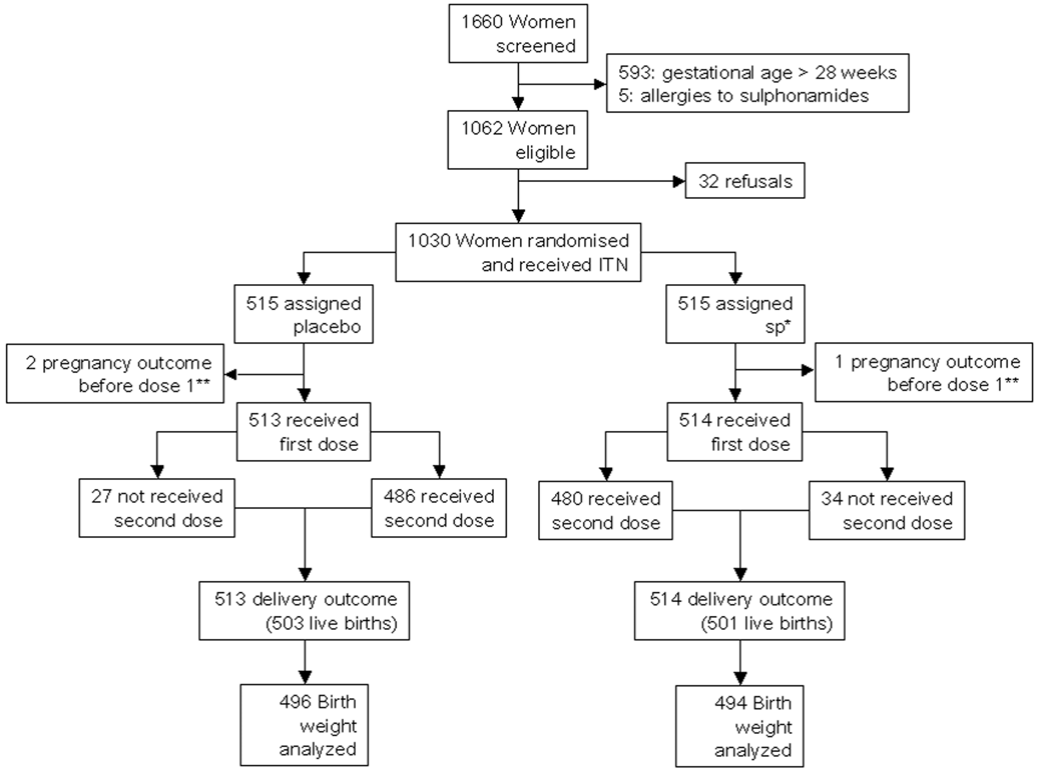
Trial profile There were 3 and 7 twins births in the SP and placebo groups respectively. There was one triplet birth in the SP Group.* sulphadoxine-pyrimethamine ** 1 woman in the SP group and 2 in the placebo group had a miscarriage before receiving dose 1

Figure 1 shows the trial profile and table 1 shows the baseline characteristics of participants. Reported ITN use at the end of pregnancy was similar between the groups (90.38% and 92.52% in the SP and placebo groups, respectively, p = 0.324). 85% of the women had an HIV test and 23.6% were HIV positive. Mean gestational age was 23.21 (SD 3.44) weeks at the first IPTp SP/placebo dose and 28.34 (SD 3.65) weeks at the second IPTp dose. Mean time between first and second IPTp SP/placebo dose was 36.53 (SD 11.55) days, and mean time between last dose and delivery was 76.91 (SD 31.19) days.

**Table 1:** Characteristics of the study women at recruitment.

		Placebo		SP*		
		(n = 515)		(n = 515)		
		Mean	(SD) †	Mean	(SD) †	p-value
**Age** (years)		24.31	(6.55)	24.00	(6.60)	0.366
**Parity**		3.12	(2.00)	3.09	(1.99)	0.809
**Height** (cm)		158.00	(5.63)	157.24	(5.99)	0.030
**Haemoglobin** (gr/dl)		10.93	(1.21)	10.97	(1.26)	0.742
		**n**	**(%)**	**n**	**(%)**	
**Gestational age** (weeks) ‡	**1st term**	4	(1)	3	(1)	0.294
	**2nd term**	291	(57)	315	(61)	
	**3rd term**	220	(43)	197	(38)	
**Gravidity**	**primigravidae**	129	(25)	137	(27)	0.821
	**1 to 3 pregnancies**	205	(40)	204	(40)	
	**4 or > pregnancies**	181	(35)	174	(34)	
**HIV test**	**negative**	338	(66)	324	(63)	0.107
	**positive**	91	(18)	117	(23)	
	**not done** §	86	(17)	74	(14)	
**MUAC index** (cm) ¶	**normal >22**	396	(98)	401	(98)	0.475
	**≤22**	10	(2)	7	(2)	
**Literacy**	**reads and/or writes**	212	(41)	213	(41)	0.346
	**neither reads nor writes**	300	(58)	302	(59)	
	**unknown**	3	(1)	0	(0)	
**RPR Syphilis test** **	**positive**	72	(14)	50	(10)	0.034
	**negative**	442	(86)	465	(90)	
	**unknown**	1	(0)	0	(0)	

* sulphadoxine-pyrimethamine

† stardard deviation

‡ 1st term: 0–12 weeks, 2nd term: 13–24 weeks,3rd term: 25–40 weeks

§ Refused voluntary testing

¶ Mid-upper arm circunference

** Rapid plasma reagin

### Safety and morbidity

Only one woman from the SP group vomited after IPTp dose 1. There were no differences in the incidence of hospital admissions and outpatient visits during pregnancy or postpartum period, or in infant visits until age 8 weeks. Nine women, similarly distributed between the groups, had mild skin reactions within 27 days after any IPTp dose (table 2). No severe skin reactions were reported. There was one maternal death (SP group), who died of obstetric causes. There were 6 infants deaths in the SP group and 11 in the placebo group (p = 0.089). There was one newborn with a major congenital malformation (spina bifida) in the SP group.

**Table 2:** Safety of SP* during the study †.

	Placebo+ITNs		SP*+ITNs				
Safety in the mother	episodes	Rate	episodes	Rate	RR	(95% CI) ‡	p-value
Incidence of all hospital admissions during pregnancy	54	0.35	52	0.32	0.89	(0.61,1.30)	0.539
Incidence of outpatient visits during pregnancy	345	1.83	327	1.64	0.94	(0.81,1.10)	0.444
Incidence of all hospital admissions during post-partum	17	0.30	20	0.35	1.06	(0.56,2.03)	0.856
Incidence of outpatient visits during post-partum	38	0.68	39	0.69	0.92	(0.59,1.43)	0.697
	**n/N**	**(%)**	**n/N**	**(%)**	**total**		
Skin reactions §	4/515	(0.77)	5/515	(0.97)	9		1.000
Maternal deaths	0/515	(0.00)	1/515	(0.19)	1		1.000

	Placebo+ITNs		SP*+ITNs				
Safety in the infant	episodes	Rate	episodes	Rate	RR	(95% CI) ‡	p-value
Incidence of all hospital admissions	25	0.46	25	0.45	0.93	(0.53,1.61)	0.786
Incidence of all outpatients visits	54	1.00	64	1.16	0.77	(0.54,1.11)	0.164
	**n/N**	(%)	**n/N**	(%)	**total**		
Infant deaths	11/503	(2.18)	6/514	(1.16)	17		0.089

* Sulphadoxine-pyrimethamine

† From recruitment up to 2 months after delivery

‡ Confidence interval

§ Within 27 days after any IPTp doses

### Effect of SP plus ITNs on main outcomes

There were no significant differences between the intervention groups in either the prevalence of LBW (RR, 0.99 [95% CI, 0.70–1.39];p = 0.940) (table 3) or in mean birth weight [3033.0 (SD 477.1) in the SP group, 3003.5 (SD 522.6) in the placebo group; p = 0.637] (table 3). SP was associated with a statistically significant reduction in the prevalence of LBW in women with ≥4 pregnancies in the subgroup analysis. Similarly, there were no significant differences between the two groups in either the prevalence of prematurity (RR, 0.74 [95% CI, 0.39–1.43];p = 0.372), or the mean gestational age [39.7 weeks (SD 1.31) in the SP group, 39.5 weeks (SD 1.49) in the placebo group; p = 0.081) (table 3). Adjustment by confounding variables did not modify these results.

**Table 3:** Low birth weight and secondary fetal and neonatal outcomes. Crude and adjusted effect by gravidity and HIV status.

		Placebo+ITNs		SP*+ITNs				
		n/N	(%)	n/N	(%)	RR	(95% CI) †	p-value
**Low birth weight (<2500 gr) ** ‡								
	crude analysis	59/496	(11.90)	58/494	(11.74)	0.99	(0.70–1.39)	0.940
	adjusted by gravidity					0.96	(0.67–1.38)	0.828
	adjusted by HIV status					0.98	(0.68–1.40)	0.900
**Low birth weight (<2500 gr) by gravidity**								
	Primigravidae	25/121	(20.66)	29/133	(21.80)	1.06	(0.66–1.70)	0.824
	1 to 3 pregnancies	13/195	(6.67)	20/194	(10.31)	1.55	(0.79–3.02)	0.197
	4 or more pregnancies	21/180	(11.67)	9/167	(5.39)	0.46	(0.22–0.98)	0.038
**Low birth weight (<2500 gr) by HIV status**								
	Negative	38/327	(11.62)	34/313	(10.86)	0.93	(0.60–1.45)	0.762
	Positive	10/85	(11.76)	17/112	(15.18)	1.29	(0.62–2.67)	0.490
	Not done §	11/84	(13.10)	7/69	(10.14)	0.77	(0.32–1.89)	0.573
**Pre-term birth (<37 weeks)**		20/407	(4.91)	15/411	(3.65)	0.74	(0.39–1.43)	0.372
**Cord blood parasitaemia**		5/435	(1.15)	4/435	(0.92)	0.80	(0.22–2.96)	0.738
**Cord blood anaemia (PCV<37%) ** ¶ **		45/435	(10.34)	22/435	(5.06)	0.49	(0.30–0.80)	0.004
**Quantitative variables (Mean (SD))**								
Birth weight		3003.55	(522.69)	3033.00	(477.11)			0.637
Newborn gestational age (weeks)		39.55	(1.49)	39.70	(1.31)			0.081
Cord blood PCV (%)		44.05	(7.49)	45.06	(7.88)			0.003
**Pregnancy outcomes**		**n**	**%**	**n**	**%**			
Spontaneous abortions (birth weight<500gr)		6	1	4	1			0.753
Stillbirths (birth weight ≥500gr)		11	2	14	3			0.686
Early neonatal deaths (<8 days of life)		5	1	0	0			0.062
Perinatal deaths (Stillbirths and early neonatal deaths)		16	3	14	3			0.716
Late neonatal deaths (>8 and <29 days of life)		0	0	2	0			0.500

* Sulphadoxine-pyrimethamine

† Confidence interval

‡ Interaction between group and gravidity, p = 0.061; Interaction between group and HIV status, p = 0.682

§ Refused voluntary testing

¶ Packed-cell volume

** Interaction between group and gravidity, p = 0.626; Interaction between group and HIV status, p = 0.191

The risk of foetal anaemia was halved among women in the SP group compared to those who received placebo (RR, 0.49 [95% CI, 0.30–0.80]; p = 0.003) (table 3). A non-statistically significant reduction in the number of early neonatal deaths was found among women receiving SP (p = 0.062) (table 3).

**Figure 2:**
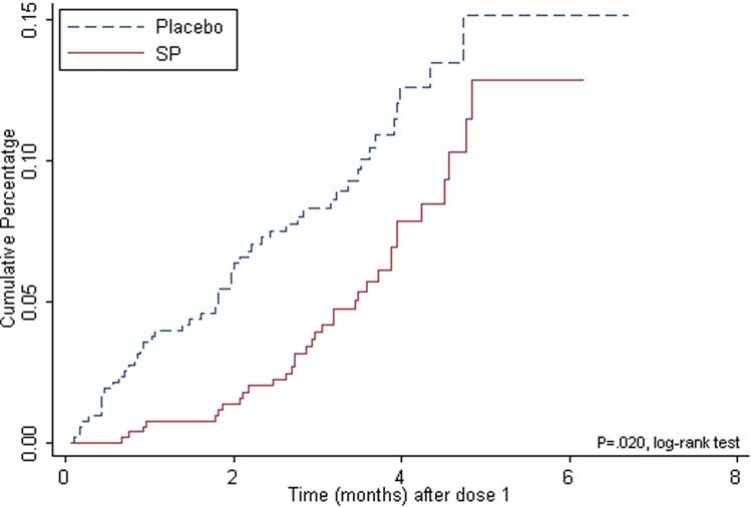
Intention to treat cohort. First or only episode of clinical malaria from IPTp dose 1 until delivery

Overall, 7% of the women receiving two-dose SP and 10% receiving placebo had a first or only episode of uncomplicated malaria (protective efficacy (PE), 40% [95% CI, 7.40–61.20]; p = 0.020) (figure 2). The analysis of the incidence of clinical malaria within the month after each SP dose showed a PE of 74.1% (95% CI, 30.8–90.3; p = 0.003) after dose 1 and of 71.4% (95% CI, 13.10–90.60; p = 0.015) after dose 2.

There was no difference in the prevalence of anaemia at delivery between the intervention and control groups (RR, 0.92 [95% CI, 0.79–1.08]; p = 0.309). However, although not significantly, there were fewer women in the IPTp/SP than in the placebo group with severe anaemia at delivery (RR, 0.25 [95% CI, 0.05–1.16]; p = 0.055). This difference was not modified by adjustments for gravidity and HIV status (table 4).

**Table 4:** Severe anaemia and parasitological outcomes at delivery. Crude and adjusted effect by gravidity and HIV status.

		Placebo+ITNs		SP*+ITNs				
		n/N	(%)	n/N	(%)	RR	(95% CI)	p-value
**Severe anaemia (PCV <21%) ** † ‡								
	Crude analysis	8/495	(1.62)	2/493	(0.41)	0.25	(0.05–1.16)	0.055
	adjusted by gravidity					0.25	(0.05–1.16)	0.077
	adjusted by HIV status					0.24	(0.05–1.13)	0.070
**Severe anaemia (PCV <21%) by gravidity**								
	Primigravidae	3/127	(2.36)	0/133	(0.00)	0.00	(.-.)	0.072
	1 to 3 pregnancies	3/193	(1.55)	1/192	(0.52)	0.33	(0.03–3.16)	0.312
	≥4 pregnancies	2/175	(1.14)	1/168	(0.60)	0.51	(0.05–5.63)	0.579
**Severe anaemia (PCV <21%) by HIV status**								
	Negative	5/324	(1.54)	1/311	(0.32)	0.21	(0.04–1.75)	0.108
	Positive	2/87	(2.30)	1/112	(0.89)	0.39	(0.04–4.25)	0.424
	Not done §	1/84	(1.19)	0/70	(0.00)	0.00	(.-.)	0.351
**Peripheral parasitaemia ** ¶								
	Crude analysis	75/495	(15.15)	35/493	(7.10)	0.47	(0.32–0.69)	0.000
	adjusted by gravidity					0.46	(0.31–0.69)	0.000
	adjusted by HIV status					0.47	(0.33–0.70)	0.000
**Peripheral parasitaemia by gravidity**								
	Primigravidae	30/127	(23.62)	18/133	(13.53)	0.57	(0.34–0.97)	0.036
	1 to 3 pregnancies	31/193	(16.06)	12/192	(6.25)	0.39	(0.21–0.73)	0.002
	≥4 pregnancies	14/175	(8.00)	5/168	(2.98)	0.37	(0.14–1.01)	0.042
**Peripheral parasitaemia by HIV status**								
	Negative	43/324	(13.27)	22/311	(7.10)	0.53	(0.33–0.87)	0.010
	Positive	17/87	(19.54)	6/112	(5.56)	0.27	(0.11–0.67)	0.002
	Not done §	15/84	(17.86)	7/70	(9.33)	0.56	(0.24–1.30)	0.165
**Any placental malaria infection ** **		219/419	(52.27)	222/426	(52.11)	1.00	(0.88–1.13)	0.964
**Positive impression smears**		57/419	(13.6)	30/426	(7.04)	0.52	(0.34–0.79)	0.002
**Positive histology**		213/419	(50.84)	218/426	(51.17)	1.01	(0.88–1.15)	0.922
**Classification of placental infection by histology**		**n/N**	**(%)**	**n/N**	**(%)**			
	acute	25/419	(5.96)	16/426	(3.75)			0.004
	chronic	51/419	(12.17)	28/426	(6.57)			
	past	137/419	(32.69)	174/426	(40.84)			
	not infected	206/419	(49.16)	208/426	(48.82)			

* Sulphadoxine-pirimethamine

† Packed-cell volume

‡ Interaction between group and gravidity, p = 0.409; Interaction between group and HIV status, p = 0.760

§ Refused voluntary counselling and testing

¶ Interaction between group and gravidity, p = 0.622; Interaction between group and HIV status, p = 0.414

** Either histology or impression smear

Table 4 shows the effect of IPTp on maternal parasitological endpoints. Peripheral parasitaemia was significantly lower among women in the SP group (7.10%, 35/493) compared to the placebo (15.15%, 75/495) (RR, 0.47 [95% CI, 0.32–0.69]; p = 0.000). This result was not modified by gravidity or HIV status.

Prevalence of any placental infection was comparable between the groups (RR, 1.00 [95% CI, 0.88–1.13]; p = 0.964), and so it was the proportion of placentas with only pigment deposition on histology (past infection). However, there were significantly less infected placentas with parasites in the SP group as assessed by impression smears (RR, 0.52 [95% CI, 0.34–0.79]; p = 0.002) or histology (acute and chronic infection) (table 4). This difference in the proportion of actively infected placentas was mainly accounted for the HIV positive women [6.12% (6/98) in the SP group versus 24.32% (18/74) in the placebo group; p = 0.001].

There were less women in the SP group than in the placebo group with malaria parasitaemia at 8 weeks after delivery (RR, 0.52 [95% CI, 0.27–0.99]; p = 0.044). There were no differences in the prevalence of women or infants with clinical malaria or anaemia (table 5). Similarly, there were no differences in the incidence of outpatient attendances or hospital admissions with malaria in either mothers or infants during the post-partum period (data not shown).

**Table 5:** Prevalence of parasitaemia and anaemia in the mother and infant at 2 months after delivery.

		Placebo+ITNs		SP*+ITNs				
		n/N	(%)	n/N	(%)	RR	(95% CI) †	p-value
**Mother**								
***P falciparum*** ** parasitaemia ** ‡								
	crude analysis	26/432	(6.02)	13/416	(3.13)	0.52	(0.27–0.99)	0.044
	adjusted by gravidity					0.50	(0.25–0.97)	0.040
	adjusted by HIV status					0.52	(0.27–1.01)	0.054
***P falciparum*** ** parasitaemia by gravidity**								
	Primigravidae	11/98	(11.22)	6/111	(5.41)	0.48	(0.18–1.25)	0.125
	1 to 3 pregnancies	9/175	(5.14)	5/161	(3.11)	0.60	(0.21–1.76)	0.351
	≥4 pregnancies	6/159	(3.77)	2/144	(1.39)	0.37	(0.07–1.79)	0.196
***P falciparum*** ** parasitaemia by HIV status**								
	Negative	20/286	(6.99)	8/263	(3.04)	0.43	(0.19–0.97)	0.036
	Positive	3/77	(3.90)	4/97	(4.12)	1.06	(0.24–4.59)	0.940
	Not done §	3/69	(4.35)	1/56	(1.79)	0.41	(0.04–3.84)	0.418
*P falciparum* parasitaemia+fever ¶		1/432	(0.23)	1/416	(0.24)	1.04	(0.06–16.55)	0.978
Overall anaemia (PCV<33%) **		89/432	(20.60)	95/416	(22.84)	1.10	(0.85–1.42)	0.461
**Infant**								
*P falciparum* parasitaemia		5/417	(1.20)	2/404	(0.50)	0.41	(0.08–2.12)	0.273
Overall anaemia (PCV<33%) **		66/417	(15.83)	63/404	(15.59)	0.98	(0.71–1.34)	0.882

* Sulphadoxine-pyrimethamine

† Confidence interval

‡ Interaction between study group and gravidity, p = 0.876; Interaction between study group and HIV status, p = 0.575

§ Refused voluntary testing

¶ Fever: T^a^ ≥37.5°C

** Packed-cell volume

## Discussion

This randomised placebo-controlled trial of IPTp in Mozambican pregnant women has shown that administering two IPTp-SP doses to women concurrently using LLITNs was safe and well tolerated, and there were no significant drug reactions despite the high prevalence of HIV among the study women. The intervention was associated with a moderate reduction in the incidence of clinical malaria during pregnancy, and with a statistically significant reduction in the prevalence of parasitaemia at delivery and at 8 weeks postpartum. However, these positive effects did not appear to translate in an improvement on relevant foetal birth outcomes such as birth weight or prematurity, nor on maternal anaemia.

It could be argued that parasite resistance to SP may explain the modest impact on some malariometric parameters and the lack of effect on the more significant birth outcomes. The available *in vivo* drug efficacy data in children in this area showed a parasitological sensitivity of 78,6% at day 14 [13], but the study was carried out 3 years before the start of the trial. Drug resistance can evolve quite rapidly, and it may be that by the time the study started, the true resistance level may have been higher. However, there is evidence that strongly suggests that SP was highly effective in the area during the study. SP was highly efficacious in participants for malaria prevention during the 30 days following each dose. A very similar effect was seen in a concurrent study of IPT in infants in the same area, whereby the efficacy of SP for malaria prevention varied between 60% and 90% in the month after SP administration [21, C. Menéndez et al., unpublished]. Furthermore, an ongoing study of placental malaria in this area has documented a sharp fall in the prevalence of placental infection following programme implementation of IPTp with SP in Mozambique (A. Mayor et al., unpublished). It is important to better understand the relationships between the assessment of *in vivo* drug resistance in sick children and the use of the same drug for prevention in asymptomatic individuals [22].

The risk of malaria during pregnancy seems to concentrate in primi and secundigravidae women [8], [23]–[27], and consequently previous IPTp studies have been carried out only in this population. There are two main reasons why we need to re-consider this approach. Firstly, targeting a specific group of women may prove difficult when scaling up this intervention to programme conditions. Secondly, our results suggest that in the presence of ITNs the effect of SP is independent of gravidity.

As previously reported, SP reduced the prevalence of malaria parasitaemia at delivery and post-partum [2], [9]. However, this reduction was not associated with a corresponding effect on the prevalence of anaemia. In this study, the prevalence of severe anaemia at delivery was much lower than expected, probably due to the effect of ITNs [14]. This might explain the insufficient power to see an effect of IPTp on this morbidity outcome. However, no effect was observed either in the prevalence of overall anaemia, despite its much higher prevalence. Further studies are needed to understand the lack of correlation between parasitological and morbidity outcomes frequently observed in malaria prevention trials during pregnancy.

The prevalence of active placental infection (i.e., containing parasites) in the SP group (7% overall and 6% in the HIV-positive women) was similar to the one reported in Kenyan and Malawian HIV-positive women who received monthly SP (7.1% and 7.8%, respectively) [23]; [26]. In these studies three or more SP doses were needed to show a comparable impact in HIV-positive and negative women. However, in these trials, ITNs were not provided as part of the study. Our data suggest that two-dose SP may protect against peripheral and placental parasitemia in HIV-positive women if they use an ITN.

Despite the fact that bednets are not traditionally used in this area, the delivery of ITNs through the ANC was well accepted and the compliance with their use was very high.

In recent years, IPTp with SP has been the cornerstone of malaria control in pregnant women living in stable transmission areas. However, the recent controversy has raised questions as to whether this was the most appropriate approach for malaria prevention in African pregnant women given the reported increased in parasite resistance to SP in some areas [22]. This has led WHO to question the adequacy of this recommendation in favour of other less well established strategies (such as indoor residual spraying) [28]. Another line of discussion recommended increasing the frequency of SP- IPTp to monthly doses to improve its efficacy. This controversy comes at a time when most endemic countries in Africa have already, or are in the process of, implementing IPTp, with all the logistical and economic efforts associated with the introduction of new public health measures in developing countries.

In conclusion, this study provides information of public health relevance in deciding the adequacy of adding IPTp when ITNs are used, and suggests that IPTp may not be required if an ITN is used. To confirm these findings, more studies of similar design are needed in areas of different malaria endemicity in Africa. In the meantime, efforts should be focused to promote the delivery of long-lasting ITNs as part of other routine ANC health interventions.

## Supporting Information

Protocol S1 Study Protocol
(0.10 MB DOC)

Checklist S1 CONSORT Checklist
(0.05 MB DOC)
