# Supplementary material for: A Randomized Placebo-Controlled Trial of Intermittent Preventive Treatment in Pregnant Women in the Context of Insecticide Treated Nets Delivered through the Antenatal Clinic
Source: PLoS One. 2008 Apr 9;3(4):e1934. doi: 10.1371/journal.pone.0001934 (PMC2277457; doi:10.1371/journal.pone.0001934)
Supplement: Protocol S1 — Study Protocol (0.10 MB DOC) [file pone.0001934.s001.doc]

EFFECT OF INTERMITTENT TREATMENT WITH SULFADOXINE-PYRIMETHAMINE PLUS INSECTICIDE TREATED NETS, DELIVERED THROUGH ANTENATAL CLINICS FOR THE PREVENTION OF MALARIA IN MOZAMBICAN PREGNANT WOMEN

**INVESTIGATORS AND INSTITUTIONS**

Clara Menendez (1) (2), Azucena Bardají (1) (2), Eusebio Macete (2), F. Xavier Gomez-Olive (1) (2) John J Aponte (1) (2), Susanna Hausmann-Muela (1), Armando Timana (3), Joana Nachaque (3), Katia Munguambe (2), Mamudo R Ismail (4), Cleofe Romagosa (5), Jaume Ordi (5), Avertino Barreto (6), Martinho Dge Dge (6), Samuel Mabunda (6), Sonia Enosse (6) Sonia Cassimiro (6), Abdul Mussa (6), Marcel Tanner (7), Pedro Alonso (1) (2)

(1) Centro de Salud Internacional. Unidad de Epidemiologia y Bioestadistica

Hospital Clinic (Universidad de Barcelona)

Barcelona, Spain

(2) Centro de Investigaçao em Saúde da Manhiça (CISM)

Manhiça, Mozambique

(3) Centro de Saúde de Manhiça, Mozambique

(4) Departamento de Anatomia Patológica. Hospital Central de Maputo

Mozambique

(5) Departamento de Anatomia Patológica.

Hospital Clinic (Universidad de Barcelona)

Barcelona, Spain

(6) Ministerio de Saude de Moçambique

Mozambique

(7) Swiss Tropical Institute

Basel, Switzerland

## **Summary**

Pregnant women are at an increased risk for malaria infection and disease. Maternal anaemia, low birth weight and prematurity are the most frequent adverse effects of the infection. Malaria control during pregnancy is recommended by maternal and child health programmes. The current WHO recommendation consists on the provision of insecticide treated nets (ITN’s) and intermittent preventive treatment. However, no information exists on their relative efficacy to reduce the deleterious effects of malaria infection during pregnancy. This information is of relevance to guide national malaria control programmes.

The objectives of the study are the following:

*General*

To contribute to the formulation of policies for the control of malaria in pregnant women.

*Specific*

1. To evaluate whether IPT in pregnancy provides any additional benefit to the protection afforded by ITNs on the following outcomes:

1. Proportion of low birth weight babies
2. Maternal anaemia at and after delivery
3. Parasite prevalence at and after delivery
4. Placental malaria infection
5. Gestational age of the newborn

2. To identify the operational and socio-cultural issues involved in the delivery to and use of ITNs by pregnant women.

3. To evaluate the cost-effectiveness of the interventions.

4. To determine the duration of the efficacy of long-lasting insecticide-treated nets against *Anopheles* mosquitos.

**5.** **To evaluate whether the administration of intermittent preventive treatment with SP in the mother has any effect on the development of naturally acquired immunity to malaria in the infant**

**(Amended September 22, 2004)**

1030 pregnant women will be enrolled at the time of their first antenatal visit to the preventive services of the Manhiça hospital, Mozambique. They will be individually randomised to receive either one treatment dose of SP or placebo at the beginning of the second trimester and during the third trimester respectively. All women will receive an ITN. Those with a gestational age greater than 28 weeks will be excluded. Maternal anaemia and parasitaemia will be assessed at delivery and 1 month after delivery. A placental tissue sample will be collected, the birth weight measured and the gestational age assessed. The prevalence of malaria infection and anaemia will be assessed one month postpartum. Infants will be followed up for one year after birth to determine their risk to malaria and its association with the prevention status of their mothers.

### Background

The first observations that malaria contributes to maternal anaemia and low birth-weight date back to the 30s of the last century (Wickramasuiya, 1937). Since then, there is much epidemiological evidence of an increased risk of pregnant women to malaria infection as compared to non-pregnant women and adult men. These observations refer not only to the African continent, where they are most abundant, but also to the Asian continent and Latin America (Brabin, 1988; McGregor, 1987; Nosten, 1991). The reasons for this increased risk are not entirely known, although there are hypotheses related to immunological factors and parasite adherence to the placenta (Fried & Duffy, 1996).

The severity of the impact of malaria both for maternal and foetal health markedly depends on the woman’s immunity against malaria. Thus, in areas with low or unstable malaria transmission, a woman’s specific immunity against infection will be low and the adverse effects of the infection more severe, ranging from cerebral malaria to maternal death in the mother and from congenital malaria to abortion and intra-uterine death of the foetus.

In areas where malaria transmission is more intense and stable, as in most parts of sub-Saharan Africa, women have generally a higher level of acquired immunity, and the consequences of the infection are less dramatic, and, at the same time, more difficult to evaluate. Independently of the area and the endemicity, malaria in pregnancy increases the risk of maternal anaemia and it is associated with intra-uterine growth retardation and prematurity. It is known that low birth weight is the most important single determinant of mortality during the first year of life. Likewise, women with severe anaemia at the time of delivery have a major risk of dying of intra- or postpartum haemorrhage, which is the principal cause of maternal death in Africa. Thus, even in women with high levels of acquired immunity malaria may contribute to maternal and infant morbidity and mortality (Menendez, 1995).

Malaria infection of the placenta is a frequent characteristic of infection during pregnancy, especially, and paradoxically, in women with a high level of acquired immunity against malaria. The parasite load of the placenta is often more intense than that detected in the peripheral blood, and it is usually accompanied by abnormalities of placental arquitecture. The most frequent histological feature is intervillous inflammation, which is sometimes massive, and which contributes to placental insufficiency and impairs intra-uterine growth (Ordi, 1998; Menendez, 2000). The proportion of newborns with low birth-weight is consistently higher in malaria endemic areas compared to non-endemic areas. In Tanzania, in the absence of maternal malaria control, the proportion of low birth weight is over 18% (Menendez, 2000).

It has been described that the risk of malaria is higher in primigravidae and secundigravidae, however our observations in the area of Manhiça, in southern Mozambique, do not show a clear association between malaria risk and parity, which would suggest the need to include all women in malaria prevention programmes. Molecular techniques of DNA detection of *Plasmodium falciparum* (PCR) confirmed this epidemiological observation: parasites were detected in samples from multiparous women, which were negative, by light microscopy (Saute, 2002).

The World Health Organization (WHO) has recommended that pregnant women who are exposed to malaria should receive antimalarial chemoprophylaxis (WHO, 1994). However, this recommendation has been shown difficult to follow because of inadequate compliance to the antimalarial regimens and limitations of regular drug distribution. This is why other preventive strategies have been explored with shorter and therefore easier administration of drugs.

This strategy consists on the administration of one antimalarial in treatment doses at predefined intervals after the beginning of the second trimester and a second dose at the beginning of the third trimester.

WHO currently recommends that in areas of stable transmission (as it is most of sub-Saharan Africa), intermittent preventive therapy (IPT) with an effective, preferably one-dose antimalarial drug be provided as part of antenatal care, starting from the second trimester onwards. At this time, sulphadoxine-pyrimethamine (SP) is the single-dose antimalarial with the best overall effectiveness for prevention of malaria in pregnancy in areas of Africa with stable *P falciparum* transmission.

Potential adverse reactions to SP, including cutaneous reactions and other potentially serious conditions have been monitored in several studies (Steketee, 1996; Phillips-Howard 1996). No evidence has been found of increased risk for serious cutaneous side effects or for increased jaundice in the newborn when SP has been delivered in the second and third trimesters.

In areas of Africa where resistance to SP is increasing, alternatives to SP- either alone or in combinations with artemisinin compounds – require urgent evaluation for use in pregnancy.

Another potential control strategy for malaria during pregnancy is the use of insecticide treated nets. In the other important risk group for malaria, children under the age of five years, ITNs showed a reduction of 60% in overall mortality (Alonso, 1991). Consistent results of mortality reduction from different parts of Africa convinced WHO to recommend ITNs for malaria control in children in Africa. In the case of pregnant women, several trials to evaluate the effect of ITNs for malaria control in pregnancy have been carried out in Thailand (Dolan, 1993), The Gambia (D’Alessandro, 1996) Kenya (Shulman, 1998) and Ghana (Brown, 2002) showing some contradictory results. Issues related to the distribution of the nets and its adequate use by the women could explain the discrepancies **in these early studies**. **However, results from a** recent large, randomised, controlled trial of ITN’s have shown a significant reduction in maternal anaemia, **parasitaemia** and low birth weight prevalence in women sleeping under impregnated nets in Kisumu, an area of high and perennial malaria endemicity in western Kenya (ter Kuile, 2003). **The relevance of these results for malaria control in pregnany have been recognised and moved WHO to recommend the provision of ITN’s to pregnant women as early in pregnancy as possible.** The use of ITN’s by pregnant women will have the added benefit of protecting their infants during the first year of life (the age at which most children in endemic areas are at highest risk for malaria).

**(Amended July 25, 2003)**

**Hypotheses:**

1. In the current context of recommendation and implementation of insecticide impregnated nets for the control of malaria, two intermittent doses of sulfadoxine-pyrimethamine (SP) during pregnancy do not provide an additional benefit in the prevention of the adverse effects of malaria during pregnancy to that of ITNs.
2. Preventive public health services for antenatal care constitute an adequate and effective channel for the distribution of ITNs to pregnant women in malaria endemic areas.

### Objectives

1. To evaluate whether two doses of sulfadoxine-pyrimethamine administrated from the beginning of the second trimester provides any additional benefit to the protection afforded by ITNs on the following parameters:

- Proportion of infants with low birth weight (<2.5 kg)
- Gestational age of the newborn
- Maternal anaemia at and 1 month after delivery
- Parasitaemia at and 1 month after delivery
- Placental malaria infection

1. To identify the operational and socio-cultural issues involved in the delivery and use of ITNs by pregnant women.
2. To assess the cost-effectiveness of the interventions.
3. To determine the duration of the efficacy of long-lasting insecticide-treated nets against *Anopheles* mosquitos.
4. **To evaluate whether the administration of intermittent preventive treatment with SP in the mother has any effect on the development of naturally acquired immunity to malaria in the infant**

**(Amended September 22, 2004)**

#### Study area and study population

The study will be carried out in the town and surrounding rural area of Manhiça, Manhiça District , Maputo Province in Southern Mozambique.

The town of Manhiça and the surrounding villages have a population of approximately 36000 inhabitants who are under continuous Demographic Surveillance System (DSS) and constitute the Manhiça study area of the Manhiça Health Research and Development Centre (CISM). Every 6 months, a census of the entire population is carried out and vital events (births, deaths, and migrations) are registered through weekly visits to village informants. Project field workers register all deliveries in the last 24 h at Manhiça Hospital and Maragra health centre daily.

The Manhiça Hospital with about 100 beds and Maragra Health Centre comprise the formal health services in the Manhiça study area.

The predominating ethnic groups in the area are Xangana and Ronga. People are mostly subsistence farmers growing cassava and maize as well as workers in an agricultural co-operative that grows bananas and rice. Some people are employed in a large sugar cane processing factory placed at Manhiça village. Houses are typically made of cane with thatched or corrugated roofs.

The total fertility rate is 5. Last figures (2001) on neonatal, infant and under-five mortality rates are 41.5 and 88.9 per 1000 live births, and 172.7 per 1000 children under five, respectively. Maternal mortality rate is over 200 per 100 000 live births (DgeDge 2002, unpublished). Average household size is 4.

*P falciparum* accounts for about 95% of malaria infections. *Anopheles funestus* is the main vector (72.3%). The average entomological inoculation rate (EIR) is 15.1 (Aranda C 2001, unpublished). Malaria transmission occurs throughout the year with marked seasonality. Drug efficacy studies in Manhiça have shown a chloroquine (CQ) parasite resistance level of about 54% (Gomez-Olive FX, 1999, unpublished). On the other hand, recent data on the efficacy of SP in children in this area have shown that 78% and 59% of the samples had cleared parasitaemia by day 14 and 28 respectively, while there was a therapeutic combined failure rate (early and late) of 17% of the children treated. The combination of SP and amodiaquine (AQ) provided a clinical efficacy of 100% (Abacassamo F 2002, unpublished). **In the light of these results the National Malaria Control Program had adopted the combination of SP and AQ as the first line treatment for uncomplicated malaria from August 2002. No specific recommendations were done regarding a policy for the treatment of uncomplicated malaria in pregnancy which remains to be defined in the country. On the other hand, there is still insufficient information regarding the safety (and efficacy) of AQ alone or in combination with SP in pregnancy (Newman et al 2003). For this reason study women with uncomplicated malaria will be initially treated with oral CQ (25 mg/kg for 3 days). Quinine will be given as a rescue treatment and in the case of severe malaria.**

**(Amended July 25, 2003)**

Bed nets are rarely used in the District of Manhiça with less than 2% of the population owing a net (not impregnated).

A recent descriptive cross-sectional study on the prevalence of malaria and anaemia in pregnant women in the Manhiça study area showed a prevalence of *P falciparum* parasitaemia of 23% and that of anaemia of 60% (Saute F, 2002).

*Number of births and antenatal attendance*

Annually, about 1500 (1521 in the year 2000) live births are registered in the Manhiça study area. Of these, about 851 take place at Manhiça hospital and 392 at Maragra health centre giving a total prevalence of health facilities deliveries of 82%.

The maternal and child health program in Mozambique currently recommends a total of 9th antenatal visits scheduled 1 month apart throughout the gestation. Women are invited to come every month up to the 28^th^ week of gestation and from then every two weeks until delivery. The gestational age at the first visit is 20 weeks on average. Most women attend the ANC services at least 3 times during pregnancy.

A sample of blood is routinely taken at the first antenatal visit to rule out syphilis infection (RPR test) and anaemia by measuring the Hb level. All women receive haematinics (ferrous sulphate and folic acid) for 1 month. Women with a haemoglobin (Hb) level less than 8g/dl are referred to the maternity ward. Although recommended by the Ministry of Health, malaria chemoprophylaxis is not a routine practice at antenatal services. Delivery of preventive supplements to pregnant women is limited to haematinics. Tetanus toxoid is administered depending on the completion of immunization status (up to 5 booster doses in total, 2 of them during gestation).

# Study design and methodology

This is a randomised double-blind placebo-controlled trial to evaluate the efficacy of two treatment doses of sulfadoxine-pyrimethamine administered at the beginning of the second trimester and during the third trimester in the prevention of the adverse effects of malaria during pregnancy, in the context of utilisation of insecticide impregnated bednets. Randomisation will be done individually.

All pregnant women attending the antenatal services at Manhiça and Maragra health centre will be invited to participate if they are permanent residents in the study area and the gestational age is **equal or** less than 28 weeks. After details of the study are explained and informed consent is given, they will be randomised to either SP or placebo. If the gestational age at this visit coincides with quickening (first noted movement of the foetus) **or the fundus is palpable (at least 13 weeks of gestation)** the first dose of SP or placebo will be given. Otherwise, they will be invited to come back coinciding with the next antenatal appointment to receive the intervention. All women will receive an impregnated net and details about its use will be explained.

At the time of recruitment, parity, gestational age, maternal weight and height and demographic and socio-economic data will be recorded. Results of the RPR test, the Hb level and the subsequent treatment will be also recorded. An identity card with a unique identification number will be given. Blood will be collected to assess HIV status after voluntary counselling and testing has been done. Women who were found to be HIV positive will be encouraged to deliver at the hospital as well as offered the administration of Nevirapine for both herself and her infant.

The women will be followed up through the demographic surveillance system in place in the area. The second dose of SP/placebo will be given coinciding with the next ANC **visit at least one month apart from the previous dose.**

**(Amended July 25, 2003)**

At the time of delivery, 5 ml of venous blood will be collected into EDTA tubes and two thick blood smears and filter paper prepared from the woman and cord blood for haematological determinations and parasitological examination. The weight of the newborn will be measured and the gestational age assessed by the Dubowitz’s method.

The placenta will be placed in a plastic container with 50 ml of 50mM EDTA in phosphate buffered saline to prevent clotting and kept in a cool box until its transport to the Center’s lab.

One month after delivery the woman and infant will be seen either at the EPI clinic coinciding with the first routine immunization visit of the newborn or at home. At this visit, the axillary temperature will be recorded and a capillary blood sample collected from both the mother and infant for haematological (haematocrit) determination and parasitological (thick blood smear and filter paper) examination. Those women or infants with fever and malaria parasites in the blood smear will receive antimalarials according to the national guidelines.

Women who deliver at home will be visited within one week of delivery. At this visit a capillary blood sample will be taken into an EDTA microtainer, a thick blood smear and filter paper collected from both mother and infant and the weight of the baby measured. Again the axillary temperature will be recorded. The newborn’s gestational age will be assessed following a simplified method.

**One year after delivery the woman and infant will be invited to come for a follow up visit and a capillary blood sample will be collected in the infant for haematological (haematocrit), parasitological (smear and filter paper), and immunological (microtainer) evaluations.**

Incidence of malaria episodes up to one year of age will be recorded in the cohort of infants through the passive case detection system established at the health facilities in the study area. Demographic events such as migrations and deaths will be documented through the DSS.

All along the trial, the study cohort (mothers and their infants) reporting sick either at Manhiça or Maragra health centres will be seen by project personnel. Standardise procedures including the study subject identification, recording the axillary temperature, administration of a standardise questionnaire and a physical exam will be carried out. Patients with fever or history of fever in the last 24 hours or who appear pale will have a thick and thin blood film done and a haematocrit collected. In case of malaria parasitaemia or anaemia they will be treated following national guidelines.

**In a subsample of approximately 200 mother and child pairs, a capillary blood sample (microtainer) will be collected in the infant at 3 and 9 months during the visit to the EPI clinic to assess the development of immune responses to malaria during the first year of life**

**(Amended September 22, 2004)**

*Exclusion criteria*

Women who are not permanent residents in the Manhiça study area or whose gestational age at the first antental visit is greater than 28 weeks will be excluded. Those who refer a history of allergy to sulfa drugs will also be excluded.

*Interventions and randomisation procedures*

SP (Fansidar®) and its placebo will be provided by Hoffman La Roche. ITN’s will be provided by UNICEF Mozambique.

Women will be individually randomised to receive either SP or placebo. Treatment identification letters will be randomly ordered in blocks of ten by a computer-generated list. Women will be individually allocated to a sequential study number that would be the lowest available identification number in a list. The next available letter will be assigned to the woman.

**Community perspectives of malaria and its control during pregnancy**

Acceptance and compliance with malaria control during pregnancy depends among other things, on the awareness of malaria as a disease and the knowledge about signs and symptoms of malaria. Particularly important is whether malaria is recognised as dangerous during pregnancy. Recognition of malaria is also related to the ability of women to distinguish signs of malaria from those of other health problems. The latter will be explored in the context of this study.

Prevention is one of the most important strategies for malaria control in pregnancy. The combination of different prevention strategies is a new proposal for improving malaria control in pregnancy.

Understanding the use of bednets as a preventive tool against malaria by the population cannot be assumed. Perceived risk of contracting malaria and perceived severity are likely to influence bednet use. The link between malaria prevention and ITN use by pregnant women will be examined in this study.

Although pregnant women will receive free ITNs at the antenatal clinic, it is not clear whether they have the power to decide about their use within the household. If there are other persons relevant for decision-making, to what extent do they recognise the importance of preventing malaria in the pregnant woman, and to what extent is priority given to the use of bednets by pregnant women?

One of the major open questions is to what extent ITNs really reach the population to which they were originally targeted. Providing free nets to pregnant women bears the danger of people selling them instead of using them themselves. High leakage rates might challenge the delivery of free nets. Since this study is set within a controlled trial design, it is not possible to adequately assess leakage rates. However, observations about leakages will be recorded as they can be very informative for later implementations.

Similarly, although intermittent preventive treatment has the great advantage that it can be administered as a single dose at the antenatal clinics, thus reducing problems of compliance, it is not clear to what extent it is accepted by the women. The latter will be also investigated within the current study.

Further details of this ancillary study are explained in Annex 1.

**Cost-effectiveness assessment of the intervention**

Once the efficacy of the interventions has been established, the following objective will be studied:

- The assessment of the cost-effectiveness ratios, of intermittent preventive treatment and/ or ITNs in the prevention of malaria and severe anemia at the end of the pregnancy.

The comparisons will include the perspectives of the financial costs to the Ministry of Health and the families and the non-health costs to the families.

A. Cost measurements

The costs will include the following:

1. Cost of the intervention within the mother and child health (MCH) preventive program for the Ministry of Health.
2. Cost of the intervention for the families will be assumed to be insignificant since the intervention visits will be linked to those for the MCH visits.
3. Cost of treatment of malaria and anemia in pregnant women for the Ministry of Health. The direct cost of an episode of malaria or anemia is mainly due to the treatment itself (drugs, blood transfusion, consultations, and tests). The cost per treatment will be separately assessed for outpatient visits and hospitalized patients, to estimate the cost-effectiveness according to the severity of disease.
4. Social cost of sickness for the family, direct and indirect costs of illness (eg travel charges, productive time lost). This will be estimated locally by a survey to be carried out as part of the economic assessment of the intervention. The cost of the adult caring for the sick pregnant woman will be estimated by calculating the lost productivity during the sickness episode as a proportion of the GDP per capita (Sauerbon et al 1991) (Tanner & Vlassoff, 1998).

The economic costs of losses for society (eg the cost of a death, including immediate (funeral) expenses and longer term lost productivity) will not be included in this cost effectiveness assessment as the study does not have sufficient power to address this endpoint. This will avoid deaths being counted twice, as such losses are likely to be included in the effectiveness estimates.

B. Effectiveness measurement

The outcomes measured for clinical malaria episodes will be:

- Years of life lost due to clinical malaria (YLL), discounted at 3%
- Healthy days of life lost due to clinical malaria episodes (all episodes)
- DALYs due to clinical malaria (YLL + YLD)

The outcomes measured for severe anemia will be:

- Years of life lost due to severe anemia (YLL), discounted at 3%
- Healthy days of life lost due to severe anemia (all episodes) (YLD)
- Disability adjusted life years (DALYs) due to severe anemia (YLL + YLD)

Methodological outline for DALY estimation

Time is the unit of measurement for the DALY. This includes time lost from premature mortality (YLL) and from morbidity (YLD).

Time lost due to premature mortality (YLL) is calculated from the age at death and life expectancy at that age. Accordingly, with the DALY methodology, the approach used will be 'standard years of life lost'. These calculations are based on standard model life tables West 26, with a life expectancy of 82.5 years for women; and standard model life table West 25 for men', with a life expectancy of 80 years (Murray, 1995). With this method, deaths at all ages will be considered as a loss and therefore included in the analysis, with a slight difference in the standard for males and females. Use of the cohort’s average life expectancy would make interventions in countries with low life expectancy more expensive than better off countries with high life expectancy.

The time lost due to sickness (YLD) is estimated from the incidence of anemia and any episode of clinical malaria, its case fatality rate and percentage of cases treated and not treated. Discapacity coefficients are assigned from those used by the Global Burden of Disease Study (Murray & Lopez, 1996). We have considered that each case remits, in the period for duration of illness specified above, unless complicated by death.

**Evaluation of the performance of long-lasting insecticide treated nets**

Long-lasting deltamethrin treated nets will be used. These nets have been shown to retain their efficacy against killing *Anopheles* mosquitos for longer periods than conventional insecticide treated nets in experimental studies. This may have potential public health as well as economic advantages by avoiding frequent re-treatment. The duration of the efficacy of the ITNs used in this study will be evaluated using WHO- standard bioassay tests and procedures against resistant and susceptible *Anopheles* mosquitos.

These tests will be carried out in a piece of material from a sub-sample of study nets (n= 100). In each case a new net will be replaced to its owner.

A survey to determine the actual use of the ITN by study women and the number of times the net has been washed will be carried out at specific times during the study.

##### Laboratory procedures

Thick blood smears will be stained with Giemsa’s stain and examined following standard procedures. Haematocrit will be determined in a microhaematocrit centrifuge from the blood collected into the EDTA tube and read in a Hawksley haematocrit reader. Maternal and cord blood will be separated and the plasma and the pellets stored at –70Cº.

A tissue sample (approximately 2 cm^3^ ) will be collected from the maternal surface of the placenta in an off-center position, a quarter of the distance between the umbilical cord and the edge, from an area without signs of infarction (Rasheed 1992). The biopsies will be immediately placed in 25 mL of 10% neutral buffered formalin and kept at 4ºC until their transport to the Hospital Central in Maputo where they will be processed and embedded in paraffin wax by standard techniques (Ordi 1998).

Paraffin sections 4um thick from the placental tissue, will be stained with hematoxylin and eosin, Giemsa’s stain and the periodic acid-Schiff technique. Following previous studies, placentas will be classified histologically as: i) not infected, ii) active infection and iii) past infection, depending on the presence or absence of parasites, pigment or both (Ismail 1999).

Impression smears will be prepared from the placental blood for parasitological examination. Blood from the placenta will also be collected onto filter paper for PCR determination of malaria parasites.

Molecular assays, for all blood samples, will be done on filter paper (Schleicher and Schuell nº903). A section corresponding to approximately 1/8^th^ of the blot on the filter paper, corresponding to approximately 6 µl of blood will be cut. This piece will then be washed with distilled water and placed in a PCR reaction tube. A nested PCR will be done as described by Felger (1993).

**Immunological assays in a subsample of mother-child pairs will include determination of antibody and cell-mediated immune responses specific to a selection of *P. falciparum* antigens.**

**(Amended September 22, 2004)**

##### Statistical considerations

Endpoints

Primary

Prevalence of low birth weight babies

Secondary

1. Prevalence of placental malaria infection by treatment group
2. Prevalence of *P falciparum* asexual maternal, peripheral parasitaemia at delivery
3. Prevalence of severe maternal anaemia (PCV level less than 21%) at delivery by group
4. Prevalence of premature babies
5. Prevalence of severe maternal anaemia (PCV level less than 21%) 1 month after delivery
6. Prevalence of overall maternal anaemia (PCV level less than 33%) at delivery and 1 month after delivery
7. Prevalence of maternal *P falciparum* asexual parasitaemia 1 month after delivery
8. **Prevalence of foetal anaemia in cord blood**
9. **Prevalence of *P falciparum* parasitaemia in cord blood**
10. **Incidence of malaria episodes in the first year of life**
11. **Incidence of severe anaemia (PCV less than 25%) during the first year of life**
12. **Difference in the levels of antibodies to *P. falciparum* antigens in infants between the two groups**
13. **Difference in the pattern of cellular immune responses to *P. falciparum* antigens in infants between the two groups**

The primary analysis will be done by intention to treat. Secondary analysis will only include women who took the two IPT doses and slept for at least 12 weeks under the ITN. Analysis will be also done by parity and adjusted for the gestational age at the time of starting each intervention.

###### Definitions

- Low birth weight is defined as a birthweight less than 2.5 kg.
- Premature birth is defined as a gestational age less than 37 weeks.
- Malaria infection is defined as the presence of asexual *P falciparum* parasites of any density in a blood smear **(or detected by PCR in a filter paper sample).**
- Fever is defined as an axillary temperature equal or greater than 37.5ºC.
- Clinical malaria is defined as malaria infection plus fever.
- Placental malaria infection is defined as the presence of parasites in a blood smear and/or the presence of parasites or pigment or both in placental tissue.
- Severe maternal anaemia is defined as a haematocrit less than 21% (Hb level less than 7 g/dl) in the mother.
- Severe anaemia in the infant is defined as a haematocrit less than 25% (Hb level less than 8 g/dl) in the infant.
- Overall anaemia is defined as a haematocrit less than 33% (Hb level less than 11 g/dl) in both mother and infant.
- Foetal anaemia is defined as a Hb level less than 12.5 g/dl or haematocrit less than 37% in cord blood.

**(Amended July 25, 2003)**

**(Amended September 22, 2004)**

*Sample size*

Assuming a proportion of low birth weight babies of 20% in the absence of malaria control measures and a 25% reduction in this figure by the use of ITNs to 15%, 411 pregnant women in each intervention group will be required to show a lack of difference between the two groups with a confidence interval of 7 (from 8 to 22%) at the 5% level of significance and with a statistical power of 80%, according to the formula for equivalent studies.^*^ To allow for 20% losses of individuals during the follow-up, 514 pregnant women need to be enrolled in each study group (total number 1028). Approximately 1000 pregnancies are registered per year in the study area. Assuming 5% refusals and exclusions, the estimated duration of enrolment will be 13 months.

**Ethical considerations**

The current WHO recommendation for malaria control during pregnancy in areas of stable malaria transmission consists on the provision of ITN’s and IPT with SP. However, the evidence on the efficacy of these two malaria control tools is based on studies that used only one of each intervention. It remains to be seen whether there is an additive effect of combining both of them. This is of relevance especially with respect to the use of IPT since if no significant added beneficial effect was observed it will save the potential problems of drug toxicity and resistance as well as of those of economic cost.

The use of a placebo is justified since it is the only way of assessing the additional effect of IPT with SP to that of ITN’s. Pregnant women in the placebo group will still be protected by the use of ITN’s. All women will be treated for malaria and anaemia episodes as required. This will be improved as a result of a closer monitoring from the health personnel and greater awareness from the women themselves.

In: Pocock SJ 1993. Clinical trials: A practical approach

- n= (2p (100-p)/d^2^) x f (α, β).
- where p is the expected proportion; d is the width of the interval; f is a function that depends on the power, thus for α= 0.05 and β= 0.2 is 7.9

**Trial flow chart**

**References**

1. Brabin B. An analysis of malaria in pregnancy in Africa. Bull WHO 1983; 61: 1005-1016.
2. D'Alessandro U, et al. The impact of a national bed net programme on the outcome og pregnancy in primigravidae in The Gambia. Trans R Soc Trop Med Hyg 1996; 90: 487-92.
3. Dolan G. Trans R Soc Trop Med Hyg 1994; 87: 621-626.
4. Fried M & Duffy PE. Adherence of Plasmodim falciparum to chondroitin sulfate A in the human placenta. Science 1996; 272: 1502-4.
5. Felger I, Tavul L and Beck HP. Plasmodium falciparum: a rapid technique for genotyping the merozoite surface protein 2. Exp Parasitol 1993; 77: 372-375.
6. McGregor IA. Thought on malaria in pregnancy with consideration of some factors which influence remedial strategies. Parassitologia 1987; 29: 153-63.
7. Menendez C. Malaria during pregnancy: a priority area of malaria research and control. Parasitology Today 1995; 11: 178-83.
8. Menendez C, et al. The impact of placental malaria on gestational age and birth weight. J Infec Dis 2000; 181: 1740-5.
9. Newman RD, et al. Safety, efficacy and determinants of effectiveness of antimalarial drugs during pregnancy : implications for prevention programmes in Plasmodium falciparum-endemic sub-Saharan Africa. Trop Med Int Hlth 2003; 8 (6): 488-506.
10. Nosten F, et al. Malaria during pregnancy in an area of unstable endemicity. Trans R Soc Trop Med Hyg 1991; 85: 424-9.
11. Ordi J, et al. Massive chronic intervellositis of the placenta associated with malaria infection. Am J Surg Pathol 1998; 22: 1006-11.
12. Phillips-Howard PA et al. The safety of antimalarial drugs in pregnancy. Drug Saf 1996 Mar; 14 (3): 131-145.
13. Rasheed FN, et al. Isolation of maternal mononuclear cells from placentas for use in in vitro functional assays. J Immunolo Meth 1992; 146: 185-193.
14. Saute F, et al. Malaria in pregnancy in rural Mozambique: the role of parity, submicroscopic and multiple Palsmodium falciparum infecrtions. Trop Med Inter Hlth 2002; 7: 19-28.
15. Schulman C, et al. A community randomised controlled trial of insecticide treated bed nets for the prevention of malaria and anaemia in primigravidae on the Kenyan cost. Trop Med Intern Hlth 1998; 3: 197-204.
16. Steketee RW, et al. The effects of malaria and malaria prevention in pregnancy on off-spring birth weight, prematurity, and intra-uterine growth retardation in rural Malawi. Am J Trop Med Hyg 1996; 55: 33-41.
17. ter Kuile FO, et al Reduction of malaria during pregnancy by permethrin-treated bed nets in an area of intense perennial malaria transmission in Western Kenya. Am J Trop Med Hyg 2003; 68 (Suppl 4): 50-60.
18. Wickramasuriya, GAW. (1937) In malaria and ankilostomiasis in pregnant women, ed. Wickramasuriya, GAW. pp. 5-90. London: Oxford University Press.
19. Worl Health Organisation. Antimalarial drug policies: Treatment of uncomplicated malaria and management of malaria in Pregnancy. Document WHO/MAL/ 94.1070. 1994; Geneva. WHO.
